# Deaths of Physicians by Cholera

**Published:** 1849-08

**Authors:** 


### EDITORIAL DEPARTMENT.

*Deaths of Physicians by Cholera.*—The fearful pestilence now raging in different parts of the country, has made sad inroads into the ranks of the medical profession of St. Louis, seventeen practitioners having fallen victims to the disease. The greatly increased amount of labor and anxiety incident to the prevalence of an epidemic disease, it would seem, must render Physicians more susceptible than others to the influence of the special cause; but, frequently, the immunity of this class furnishes occasion for common remark. We know of no especial reasons for their exemption, except, perhaps, greater prudence in matters under their control, and an absence of that apprehension which is so apt to pervade communities afflicted by a pestilence, and which is doubtless one of the most powerful of predisposing causes. In St. Louis, however, the severity of the epidemic, and the extent to which it has prevailed, must have imposed duties upon the medical profession, greatly disproportionate to ordinary powers of endurance, and it is probably to this fact that the mortality among its members is to be attributed. As regards contagion, we make no account of it in this, or any other connection, for reasons which we will not now discuss, but which, in our view, are sufficiently conclusive. Excluding all idea of communicability, the active exercise of medical practice, during the prevalence of a pestilental disease, involves peculiar dangers incident to toil, anxiety, loss of sleep, and constant exposure to deleterious influences. But, notwithstanding this, how seldom do medical practitioners flee from the post of duty under such circumstances! Whatever may be the place which the medical profession holds in public estimation, there is much in the character of its worthy members to gratify an honest professional pride, and to excite a noble spirit of emulation!

